# Serotype IV and Invasive Group B *Streptococcus* Disease in Neonates, Minnesota, USA, 2000–2010^1^

**DOI:** 10.3201/eid1904.121572

**Published:** 2013-04

**Authors:** Patricia Ferrieri, Ruth Lynfield, Roberta Creti, Aurea E. Flores

**Affiliations:** University of Minnesota Medical School, Minneapolis, Minnesota, USA (P. Ferrieri, A.E. Flores);; Minnesota Department of Health, St. Paul, Minnesota, USA (R. Lynfield);; Istituto Superiore di Sanità, Rome, Italy (R. Creti)

**Keywords:** bacteria, group B Streptococcus, GBS, neonatal GBS disease, invasive GBS disease, serotype IV, early-onset GBS disease, late-onset GBS disease, clindamycin resistance, streptococci, antimicrobial resistance, serotype, neonates, Minnesota, United States, capsular polysaccharide, CPS, prophylaxis

## Abstract

Serotype predominance has shifted, and drug resistance is emerging.

## Medscape CME Activity

Medscape, LLC is pleased to provide online continuing medical education (CME) for this journal article, allowing clinicians the opportunity to earn CME credit. 

This activity has been planned and implemented in accordance with the Essential Areas and policies of the Accreditation Council for Continuing Medical Education through the joint sponsorship of Medscape, LLC and Emerging Infectious Diseases. Medscape, LLC is accredited by the ACCME to provide continuing medical education for physicians.

Medscape, LLC designates this Journal-based CME activity for a maximum of 1 *AMA PRA Category 1 Credit(s)*^TM^. Physicians should claim only the credit commensurate with the extent of their participation in the activity.

All other clinicians completing this activity will be issued a certificate of participation. To participate in this journal CME activity: (1) review the learning objectives and author disclosures; (2) study the education content; (3) take the post-test with a 70% minimum passing score and complete the evaluation at www.medscape.org/journal/eid; (4) view/print certificate.

**Release date: March 15, 2013; Expiration date: March 15, 2014**

## Learning Objectives

Upon completion of this activity, participants will be able to:

- Describe the classification of infant group B *Streptococcus* (GBS) disease and the impact of maternal screening on this illness
- Analyze the epidemiology of infant GBS disease in the current study
- Distinguish the most common GBS genotypes among infants infected in the current study
- Evaluate the significance of GBS genotype IV

## CME Editor

**Shannon O’Connor, ELS, **Technical Writer/Editor, Emerging Infectious Diseases. Disclosure: Shannon O’Connor has disclosed no relevant financial relationships.

## CME Author

**Charles P. Vega, MD, **Health Sciences Clinical Professor; Residency Director, Department of Family Medicine, University of California, Irvine. *Disclosure: Charles P. Vega, MD, has disclosed no relevant financial relationships.*

## Authors

*Disclosures:* ***Patricia Ferrieri, MD; Ruth Lynfield, MD; Roberta Creti, PhD;*** *and* ***Aurea Flores*** *have disclosed no relevant financial relationships.*
