# Supplementary material for: Serotype IV and Invasive Group B Streptococcus Disease in Neonates, Minnesota, USA, 2000–2010
Source: Emerg Infect Dis. 2013 Apr;19(4):551–8. doi: 10.3201/eid1904.121572 (PMC3647718; doi:10.3201/eid1904.121572)
Supplement: Related texts

# Research article: Serotype IV and Invasive Group B *Streptococcus* Disease in Neonates, Minnesota, USA, 2000–2010^1^

**Authors:** Patricia Ferrieri, Ruth Lynfield, Roberta Creti, Aurea E. Flores

**Affiliations:** University of Minnesota Medical School, Minneapolis, Minnesota, USA (P. Ferrieri, A.E. Flores);; Minnesota Department of Health, St. Paul, Minnesota, USA (R. Lynfield);; Istituto Superiore di Sanità, Rome, Italy (R. Creti)

## Abstract

Group B *Streptococcus* (GBS) is a major cause of invasive disease in neonates in the United States. Surveillance of invasive GBS disease in Minnesota, USA, during 2000–2010 yielded 449 isolates from 449 infants; 257 had early-onset (EO) disease (by age 6 days) and 192 late-onset (LO) disease (180 at age 7–89 days, 12 at age 90–180 days). Isolates were characterized by capsular polysaccharide serotype and surface-protein profile; types III and Ia predominated. However, because previously uncommon serotype IV constitutes 5/31 EO isolates in 2010, twelve type IV isolates collected during 2000–2010 were studied further. By pulsed-field gel electrophoresis, they were classified into 3 profiles; by multilocus sequence typing, representative isolates included new sequence type 468. Resistance to clindamycin or erythromycin was detected in 4/5 serotype IV isolates. Emergence of serotype IV GBS in Minnesota highlights the need for serotype prevalence monitoring to detect trends that could affect prevention strategies.

*Streptococcus agalactiae*, or group B *Streptococcus* (GBS), is one of the leading causes of invasive bacterial diseases, such as bacteremia, pneumonia, and meningitis, in newborns and infants in the first months of life in the United States (*1*,*2*) and in other parts of the world (*3**–**6*). In newborns birth through 6 days of age, invasive GBS disease is designated as early-onset (EO) and late-onset (LO) in infants 7 days to 3 months of age; some investigators report an ultra-late period extending well beyond 3 months of age (*3*).

During the past 20 years, prevention of EO and LO invasive GBS disease in the United States has been an area of interest for clinicians and public health officials. In the early 1990s, the overall rate of EO disease in the United States was 1.7/1,000 live-born infants (*7*,*8*) but differed from one part of the country to another: for example, 0.56 for Minneapolis/St. Paul, 1.3 for Houston (*9*), and 1.3 for Maryland (*10*). This high rate prompted the Centers for Disease Control and Prevention (CDC) to issue guidelines in 1996 for preventing EO disease by screening pregnant women at 35–37 weeks’ gestation for GBS colonization and administering antimicrobial drug prophylaxis to women at risk of transmitting the organism to the child (*11*). Although implementation of the prescribed measures reduced the rate of EO disease in the United States to 0.47/1,000 live births by 1999–2001, problems remained (e.g., risk-based vs. culture-based approaches to prevention, laboratory detection of colonized mothers, use of antimicrobial drugs in women with allergies to penicillin, use of secondary prevention among infants) (*12*); these factors required revision of the guidelines in 2002 (*7*) and again in 2010 (*13*). However, measures designed to prevent EO disease have had little effect on the rate of LO disease, which remained 0.4/1,000 live births throughout the 1990s, varying only slightly from year to year (*8*,*12*). 

GBS isolates are characterized according to capsular polysaccharide (CPS) serotype, of which 9 are recognized: Ia, Ib, II–VIII (*9**, **14**–**16*), and a recently proposed serotype IX (*17*). Results from earlier studies in various parts of the United States, including Minnesota, indicated that Ia, III, and V were the predominant serotypes in EO disease, whereas serotype III and Ia were predominant in LO disease (*9*,*10*,*12*,*15*).

Since 1995, our laboratory has collaborated with the Minnesota Department of Health (MDH) to serotype isolates from cases of EO and LO disease in Minnesota in conjunction with the CDC Emerging Infections Program (*7*). This collaboration has enabled us to follow for almost 2 decades changes in serotype distribution of GBS isolates that cause invasive disease in Minnesota. Here we report on the epidemiology of EO and LO GBS disease in Minnesota over 11 years, the recent emergence of invasive disease in infants with serotype IV GBS, and an increase in disease caused by this serotype in 2010 compared with our previous findings (*15*). We provide serotyping results of all isolates from EO and LO disease collected during 2000–2010 and present data from molecular characterization by pulsed-field gel electrophoresis (PFGE) and multilocus sequence typing (MLST) of serotype IV isolates from EO and LO disease. 

## Materials and Methods

### Study Design

As part of the CDC Active Bacterial Core Surveillance Program, GBS isolates from 453 infants in Minnesota with invasive GBS disease reported during January 1, 2000–December 31, 2010, were submitted to the MDH Public Health Laboratory, and the case details were reviewed (*7*). Infants were classified according to GBS disease onset and age: 257 (age birth–6 days) with EO disease, 180 (age 7–89 days) with LO disease, and 12 (age 90–180 days) with delayed LO disease. Four infants with first GBS-positive culture at age 6–13 months were classified with ultra-LO disease (ULOD) (*3*) and were not included in our analyses. The total numbers of live births in Minnesota by year were provided by MDH.

### Study Isolates and Culture Sites

Of the 449 EO and LO isolates studied and analyzed, 403 were from blood, 42 from cerebrospinal fluid (CSF), and 4 from normally sterile sites (1 bone, 1 joint, and 2 obtained postmortem from liver and lung). One isolate per infant was included in the analysis. Isolates received from MDH without patient identifiers were serotyped and studied by molecular methods at the GBS Molecular Reference Laboratory (University of Minnesota Medical School Twin Cities Campus, Minneapolis, MN, USA). Isolates were tested by using single-colony picks in Todd-Hewitt Broth (THB) (Bacto; Becton, Dickinson and Company, Sparks, MD, USA), supplemented with 2% sheep blood.

### Reference Strains and Antiserum for Serotyping

We used GBS prototypes from our laboratory of internationally recognized reference strains of serotypes Ia, Ib, II–VIII, and newly proposed IX; Rabinowitz 3139 was used for serotype IV (*17**–**19*). The prototype strains for surface-expressed proteins were those used previously (*18*). Monospecific rabbit antisera to serotypes Ia, Ib, II–VIII, proposed IX, and atypical V (serotype V genetic variant); surface proteins C-α and C-β; group B protective surface (BPS) protein; and the R4(Rib), R1, and R1, R4 (Alp3) species of R were produced in our laboratory against international reference strains (*17*,*18*,*20*,*21*). All antisera were tested against all serotypes to ensure no immunologic cross-reactions. In addition, molecular typing by PCR confirmed all prototype strains used for production of each type-specific antiserum, including serotype IV.

### CPS and Detection of Surface-expressed Proteins

Isolates grown in THB were extracted with hot hydrochloric acid and typed by double-diffusion immunoprecipitation in agarose slides with specific antisera to serotypes Ia, Ib, and II–VIII, as described (*18*). An isolate nonreactive with any of these antisera was considered nontypeable and was regrown in supplemented THB to upregulate CPS production; the extract was retested (concentrated 3-fold) with antisera to Ia–VIII, proposed IX, and atypical V. Extracts from nontypeable and serotype IV isolates were also tested with antisera to GBS surface-expressed proteins (*16*,*18**–**20*). Statistical analyses of the typing results (2-sided p value, Fisher exact test) were done by using InStat (GraphPad Software, Inc., San Diego, CA, USA); p values <0.05 were considered significant.

### DNA Macrorestriction Profile Analysis

All 12 serotype IV and 2 nontypeable isolates were studied by PFGE according to published methods (*16*,*22*,*23*) by using bacterial DNA digested with *Sma*I (Invitrogen, La Jolla, CA, USA). The PFGE profile of an isolate was determined by comparing its macrorestriction band pattern to those of the prototype isolates in our PFGE library (*16*,*22*), including the prototypes from 4 PFGE profile groups delineated among our serotype IV isolates from recent years.

### MLST and Clonal Complex Assignment

At least 1 serotype IV isolate from each PFGE profile was studied by MLST as described (*14*,*16*,*24*). Also studied were 4 serotype IV blood isolates collected from 2 mothers during the peripartum period and from 2 nonpregnant adults. Results from partial sequencing of 7 housekeeping genes were compared with data from the *Streptococcus agalactiae* MLST Database (http://pubmlst.org/sagalactiae/) to arrive at an allelic profile and sequence type (ST) for each isolate, as described (*16*). The clonal complex (CC) assignment of each isolate was determined by using eBURST software (*25*) so that each CC comprised STs that had alleles at 6 of 7 loci in common with >1 other member of the complex. The study STs were compared with all STs in the *Streptococcus agalactiae* MLST Database.

### Antimicrobial Drug Susceptibility Studies

All 45 isolates from 2010 were tested for susceptibility to clindamycin and erythromycin according to Clinical and Laboratory Standards Institute guidelines (www.microbiolab-bg.com/CLSI.pdf) by a microdilution MIC method or gradient diffusion E-test. For 2000–2009, only the 7 serotype IV isolates were tested. Isolates sensitive to clindamycin but resistant to erythromycin were further tested by using a double-disk diffusion D-zone test to determine whether resistance to clindamycin could be induced (*26*).

## Results

During January 2000–December 2010, a total of 257 infants in Minnesota with invasive GBS disease had EO disease, with GBS isolated from cultures taken during the first 6 days of life (Table 1). An additional 192 infants had LO disease, 180 with GBS-positive cultures at 7–89 days and 12 at 90–180 days of age (delayed LO). Four isolates from infants with ULOD were not included in our analyses. Nearly 90% of all isolates were from blood, but the source of the isolates differed significantly for EO versus LO disease: 251 EO isolates were from blood, compared with 152 LO isolates (p<0.0001), but CSF was the culture source for 4 EO isolates compared with 38 LO isolates (p<0.0001).

**Table 1:** Distribution of GBS invasive disease in infants by isolate source and age of infant at time of culture, Minnesota, USA, 2000–2010*

Culture source	No. infants with GBS invasive disease†				Total
	Early onset	Late onset			
		7–89	90–180	>181	
Blood	251	142	10	3	406
Cerebrospinal fluid	4	37	1	1	43
Other sites‡	2	1	1	0	4
All	257	180	12	4	453

*GBS, group B Streptococcus. †No. isolates studied were 1 per infant (n = 453). Early-onset is birth–6 days. Late-onset categories (age in days at time of culture): classical (7–89); delayed (90–180); ultra (>181; not included in analyses). ‡Other sites, normally sterile: 1 bone, 1 joint, 2 tissues (1 liver, 1 lung).

III and Ia were the predominant serotypes, accounting for approximately two thirds of the 449 GBS isolates from all EO and LO invasive disease (Table 2). Nearly another third consisted of serotypes V, II, and Ib. A few isolates, mostly from infants with EO disease, were serotype IV or VII; 5 (1.1%) were nontypeable. All serotype IV isolates were identified by routine serotyping and did not require supplemental growth or concentration of the extracts; only a few isolates of other serotypes required these extra approaches.

**Table 2:** Serotype distribution of invasive GBS isolates for cultures collected from infants, Minnesota, USA, January 2000–December 2010*

CPS type	No. (%) patients†		
	Early onset	Late onset	Total
Ia	79 (30.7)	57 (29.7)	136 (30.3)
Ib	25 (9.7)	12 (6.2)	37 (8.2)
II	43 (16.7)	5 (2.6)	48 (10.7)
III	57 (22.2)	95 (49.5)	152 (33.9)
IV	8 (3.1)	4 (2.1)	12 (2.7)
V	39 (15.2)	18 (9.4)	57 (12.7)
VII	2 (0.8)	0 (0.0)	2 (0.4)
Nontypeable	4 (1.6)	1 (0.5)	5 (1.1)
Total	257 (100.0)	192 (100.0)	449 (100.0)

*GBS, group B Streptococcus; CPS, capsular polysaccharide serotype. †Early-onset, patient age birth–6 days; late-onset, patient age 7–180 days.

When the distribution of serotypes in EO versus LO disease was compared, Ia was most commonly isolated in the 257 EO cases (79, 30.7%), followed by III (57, 22.2%), II (43, 16.7%), and V (39, 15.2%). In contrast, among the 192 LO isolates, serotypes III (95, 49.5%) and Ia (57, 29.7%) predominated. Serotypes Ia and IV were distributed similarly among EO and LO isolates (Ia, 30.7% of EO vs. 29.7% of LO; IV, 3.1% of EO vs, 2.1% of LO). Serotypes II and III, however, had significantly different distribution in EO and LO disease; serotype II accounted for 16.7% of EO versus 2.6% of LO isolates (p<0.0001), whereas serotype III accounted for 22.2% of EO versus 49.5% of LO isolates (p<0.0001). Of the 42 isolates from CSF, serotype III accounted for 50%, whether from infants with EO (2/4) or LO (19/38) disease. The 4 isolates from ULOD were 1 each of serotypes Ia, Ib, II, and V (data not shown).

During the 11-year study period, the number of infants with EO or LO invasive disease varied from year to year; most years had more EO than LO cases (Figure 1). This variation resulted in yearly incidence that ranged from 0.21 to 0.45 (mean 0.33) per 1,000 live births for EO and from 0.14 to 0.34 (mean 0.25) per 1,000 live births for LO disease (Figure 2). Although serotype IV was seen only sporadically in previous years (Figure 1), incidence of this serotype increased in 2010, when it was isolated from 5 (16.1%) of 31 infants with EO disease. These 5 cases were not clustered geographically or temporally.

**Figure 1:** Distribution of early-onset and late-onset invasive group B *Streptococcus* disease in infants, by year, Minnesota, USA, 2000–2010. Bars indicate isolates of all capsular polysaccharide serotypes (CPS); line indicates all serotype IV isolates. A total of 257 infants had early-onset and 192 infants late-onset disease; 12 infants had type IV infection.

**Figure 2:** Incidence of early-onset and late-onset group B *Streptococcus* disease per 1,000 live births, by year, Minnesota, USA, 2000–2010.

Among the 12 infants who had serotype IV invasive disease during 2000–2010, 11 survived (Table 3). Eight infants had EO disease, all of which were diagnosed within 2 days of birth; only 4 infants, separated temporally, had LO disease. All infants with EO disease were full term (gestational age 37–42 weeks); for LO infants, all but 1 were not full term (gestational age 23–34 weeks). Overall, the proportion of babies born prematurely (<37 weeks) and infected with any GBS serotype was 23.4% (54/231) for EO disease, 47.0% (77/164) for LO disease, and 53.8% (7/13) for delayed LO or ULOD disease (data not shown).

**Table 3:** Patient characteristics of and clinical data for serotype IV GBS isolates from infants with invasive GBS disease, Minnesota, USA, January 2000–December 2010*

Period	Disease type†	Patient age at diagnosis	Patient outcome	Culture source	Clindamycin susceptibility‡	Isolate protein profile	Isolate PFGE profile
2001–2009	Late-onset	98 d	Survived	Joint fluid	S	C-α and BPS	37a
	Late-onset	30 d	Survived	Blood	S	C-α and BPS	37
	Late-onset	9 d	Died	Blood	S	BPS	37a
	Early-onset	1 d	Survived	Blood	S	None	40
	Late-onset	9 d	Survived	Blood	S	BPS	37a
	Early-onset	2 d	Survived	Blood	S	BPS	37a
	Early-onset	1 d	Survived	Blood	S	BPS	37a
2010	Early-onset	1 d	Survived	Blood	R	C-α	39a
	Early-onset	Newborn	Survived	Blood	R	C-α	39c
	Early-onset	Newborn	Survived	Blood	R	C-α	39a
	Early-onset	Newborn	Survived	Blood	R	C-α	39a
	Early-onset	Newborn	Survived	Blood	S	BPS	37a

*GBS, group B Streptococcus; PFGE, pulsed-field gel electrophoresis; C-α, C-protein α; BPS, group B protective surface protein. †Early-onset, patient age birth–6 days; late-onset, patient age 7–180 days. ‡S, susceptible, MIC <0.25 μg/mL; R, resistant, MIC >1 μg/mL.

Blood was the most common culture source for serotype IV isolates (11/12); 1 was from a joint fluid. Isolates from all 7 infants who had serotype IV GBS disease during 2000–2009 were susceptible to clindamycin, but 4/5 (80%) isolates from infants who had EO disease in 2010 were clindamycin resistant.

Emergence of resistance to clindamycin in serotype IV isolates during 2010 led us to investigate antimicrobial drug resistance for all 45 serotype IV isolates cultured from infants with invasive GBS disease during that year. Antimicrobial drug susceptibility profiles (Table 4) revealed that 14 (31.1%) of these isolates were resistant to clindamycin and erythromycin; clindamycin resistance was inducible for 4 of these 14 isolates. In addition, a higher percentage (>60%) of isolates of serotypes IV, V, or Ib were clindamycin resistant compared with isolates of the predominant serotypes Ia (8.3%) and III (11.1%). Specifically, the percentage of serotype IV isolates that were clindamycin resistant (4/5, 80%) was significantly higher than for all other serotypes combined (10/40, 25%; p = 0.027). In 2010, serotype IV accounted for 28.6% (4/14) of all isolates resistant to clindamycin.

**Table 4:** Clindamycin susceptibility profiles of GBS isolates from 45 infants with invasive GBS disease, Minnesota, USA, 2010*

Antimicrobial susceptibility profile†				CPS serotype						Total
Clindamycin	Erythromycin	D-test		Ia	Ib	II	III	IV	V	
S	S	Not done		7	1	0	13	1	2	24
S	R	Negative		4	0	0	3	0	0	7
S→R	R	Positive		1	1	0	2	0	0	4
R	R	Not done		0	2	1	0	4	3	10
Total no.				12	4	1	18	5	5	45
No. (%) resistant				1 (8.3)	3 (75.0)	1	2 (11.1)	4 (80.0)	3 (60.0)	14 (31.1)

*GBS, group B Streptococcus; CPS, capsular polysaccharide serotype. †S, susceptible, MIC <0.25 μg/mL; R, resistant, MIC >1 μg/mL; S→R, inducible resistance to clindamycin indicated by positive D-zone test (double-disk diffusion test).

Because of the increase of invasive disease caused by serotype IV GBS, we pursued molecular studies on isolates of this serotype. Figure 3, panel A, shows the PFGE DNA macrorestriction band patterns of the serotype IV prototype isolates, designated 37, 38, and 39 (*16*), and profiles we classified as 39c and 39a from 2 study isolates expressing only C-α protein. Profiles of 6 isolates with C-α protein and BPS protein or only BPS protein appeared to be identical or very similar and were designated 37 or 37a for their resemblance to the group 37 prototype (Figure 3, panel B). The profile of an isolate that did not express any of the surface proteins studied was unique and was classified as profile 40. Overall, isolates with C-α and BPS proteins or BPS protein alone were in PFGE profile group 37 or its subgroups and were susceptible to clindamycin; those with only C-α protein were in subgroups of PFGE group 39 and were resistant to clindamycin (Table 3).

**Figure 3:** DNA macrorestriction profiles for serotype IV isolates from invasive group B *Streptococcus* (GBS) disease in infants, Minnesota. Isolates were studied by *Sma*I digestion and pulsed-field gel electrophoresis (PFGE) analysis and were designated as expressing C-protein α (C-α) or group B protective surface protein (BPS). Lane number is at the top and PFGE profile number at the bottom of each lane. A) Lane 2, λ molecular size standard; lanes 3 and 4, serotype IV/C-α GBS isolates from early-onset disease; lanes 5–7, prototypes of PFGE profile groups 39 (IV/C-α), 38 (IV/C-α), and 37 (IV/C-α and BPS); lane 8, internal standard 89-022 (Ib/C-α and C-β). B) Lane 1, λ molecular size standard; lane 2, PFGE profile 37 prototype (IV/C-α and BPS); lanes 3–6, isolates from late-onset disease; lanes 7–9, isolates from early-onset disease; lane 10, internal standard 89-022. Protein profile of isolates in lanes 2–4, C-α and BPS; lanes 5–8, BPS only. The isolate in lane 9 did not express any of the proteins studied.

To investigate further genetic relatedness among serotype IV isolates from invasive GBS disease in Minnesota, we studied 4 isolates from infants and 4 from adults by using MLST (Table 5). We found that the serotype IV prototype strain 3139, studied for comparison, was ST2 in CC1 (data not shown). Among isolates from infants, 1 was ST196, 2 were ST452, and 1 was ST468, a new sequence type (allelic profile 5,25,4,3,2,3,1). Among isolates from adults, 1 was ST196, 2 were ST291, and 1 was ST459. The 5 STs were grouped into 3 major CCs: ST196 and ST459 in CC1, ST291 in CC17, and ST452 and ST468 in CC23. ST468 was a single-locus variant of ST452, which we recently described (*16*), while ST459 was a single-locus variant of the much older ST196. Of the 8 isolates, 1 (PFGE profile 39a, ST459) was resistant to clindamycin (data not shown), as were other isolates in subgroups of PFGE profile group 39 (Table 3). The PFGE profiles of all the serotype IV isolates were classified into 5 groups or their subgroups: 37, 39, and 40 for isolates from infants; 36, 38, and 39 for isolates from adults (Tables 3, 5).

**Table 5:** Molecular characteristics of serotype IV GBS isolates causing invasive disease in infants and adults, Minnesota*

Isolate source†	PFGE profile†	Allelic profile‡	Sequence type§	Clonal complex¶
Mother, early-onset	38d	1,1,3,1,1,12,2	196	1
Infant, early-onset	40	1,1,3,1,1,12,2	196	1
Nonpregnant adult	39a	1,1,3,1,41,12,2	459	1
Mother, early-onset	36c	2,25,1,2,1,1,1	291	17
Nonpregnant adult	36d	2,25,1,2,1,1,1	291	17
Infant, late-onset	37	5,25,4,3,2,3,3	452	23
Infant, late-onset	37a	5,25,4,3,2,3,3	452	23
Infant, early-onset	37a	5,25,4,3,2,3,1	468	23

*GBS, group B Streptococcus; PFGE, pulsed-field gel electrophoresis. †Early-onset, patient age birth–6 days or mother during peripartum period; late-onset, patient age 7–180 days. ‡Multilocus sequence type of 7 housekeeping genes (adhP, pheS, atr, glnA, sdhA, glcK, tkt). §From http://pubmlst.org/sagalactiae database. ¶Determined by eBurst analysis (*25*).

## Discussion

As in the rest of the United States and other parts of the world (*8*,*12*,*13*,*27*,*28*), in Minnesota, GBS is one of the leading causes of invasive bacterial infections in infants during the first year of life. Overall, the yearly incidence rate of EO and LO disease in this state decreased modestly from 2000 to 2010; however, even with implementation of control guidelines issued by CDC, some years had an increase in incidence, as occurred for EO disease in 2010.

Our finding that GBS caused invasive disease beyond the third month of life for ≈9% of infants in our study was in keeping with previous reports (*3*). Investigators have found that premature birth was a major risk factor for LO or ULOD GBS disease (*8*,*13*,*28*,*29*). Prematurity was likely a contributing factor for LO disease in infants <90 days of age, in particular for those with delayed LO disease or ULOD. The proportion of infants with these disease types who were born preterm was >2× that for infants with EO disease; in Minnesota, as in other parts of the United States (*30*), ≈75% of infants with EO disease are born at full term.

Our results on the distribution of serotypes from all GBS invasive disease were consistent with studies showing that, worldwide, serotype III continues to predominate, followed by serotypes Ia and V (*27*,*31*). We found that the predominant serotypes in EO disease continued to be Ia, followed by III, with these serotypes in reverse order for LO disease, similar to our previous findings for Minnesota (*15*) and findings for other parts of the country (*9*). However, in marked contrast to the 1990s, when the prevalence of serotype V was 2× that for serotype II (*15*), serotype II was the third most prevalent serotype for EO disease in our study.

Characterization of GBS isolates by serotype over time enabled us to track the emergence and spread of serotype IV as a cause of invasive disease in Minnesota. These results showed that this previously less common serotype has become more common, starting in the mid-1990s, when serotype IV was found in a few adults (2 mothers in the peripartum period and 1 nonpregnant adult) (*15*); subsequently, in 2001, the first case of LO disease caused by serotype IV was found, and in 2010, this serotype became prominent in causing GBS disease. Isolation of this serotype in 2011 from 2 infants with LO disease suggests its continued presence in the community (P. Ferrieri, unpub. data).

Our findings raise the possibility that serotype IV, although reported previously from a case of EO disease in the United States (*32*) and in small numbers from other parts of the world (*6*), has the potential to emerge as a notable cause of invasive GBS disease not only among newborns but also among older infants through the first year of life, just as occurred with serotype V during the past 2 decades (*33*,*34*). The sharp increase in 2010 of disease caused by serotype IV isolates and concurrent emergence of clindamycin resistance within this serotype could foreshadow problems similar to those for serotype V; a high percentage of antimicrobial drug–resistant serotype V isolates (*14*,*26*,*35*) cause disease in infants and older adults (*33*,*36*). We found not only an increase in the percentage of nonpregnant women in the United States colonized with serotype IV in vaginal/rectal sites (*16*) but also evidence of clindamycin resistance, with 3 of 8 representative serotype IV isolates studied found to be resistant (P. Ferrieri, unpub. data). Because the vaginal tract is a reservoir for GBS causing EO disease (*13*,*37*), increased colonization of this site with clindamycin-resistant serotype IV GBS is of concern. Careful attention must be given to the type of intrapartum antimicrobial drug prophylaxis administered; GBS isolates should be assessed for inducible clindamycin resistance when penicillin, ampicillin, or cefazolin cannot be used.

Among the serotype IV isolates from invasive GBS disease, we observed association among PFGE profile, surface protein profile, and susceptibility/resistance to clindamycin. Isolates with only BPS protein or C-α and BPS proteins were in PFGE group 37 and susceptible to clindamycin; those with only C-α protein were in group 39 or its subgroups and resistant to clindamycin. Results from a study of serotype IV isolates from colonized nonpregnant women living in various areas in the United States showed a similar association between surface protein profile and PFGE profile (*16*). MLST results suggested a possible association between clindamycin resistance and ST459.

In contrast to colonizing serotype IV isolates, which have limited clonal diversity (*16*), results from our molecular studies of invasive serotype IV isolates showed that the 16 isolates cultured since 1995 spanned 5 PFGE profiles, 5 STs, and 3 CCs, which suggests greater genetic diversity. Among 97 colonizing serotype IV isolates from a wider area of the United States, we found 3 PFGE profile groups (37–39) and 2 STs (452 and 459) (*16*); in contrast, among just 16 invasive isolates in this study, we found the same PFGE profiles and STs and 2 additional PFGE profile groups (36 and 40) and STs (196 and 468). In other MLST studies of GBS isolates (*4**–**6*,*14*,*38*,*39*), only small numbers of serotype IV have been included, and these have most often been placed in CC1 and a few in CC17, 2 CCs with STs seen in our study. The fact that 3 of our serotype IV isolates, particularly ST459, were in CC1 may be of importance because a large percentage of serotype V isolates, many of them antimicrobial drug resistant, have been placed in this CC (*4*,*14*). Two of our invasive isolates were in CC17 and 3 in CC23; these are well-known CCs that contain many isolates of various serotypes from invasive disease in infants and adults (*38*,*39*) or that are associated with virulent clones (*5*,*6*,*38*). ST452 and ST459 are new STs from women colonized with serotype IV (*16*).

The emergence of invasive serotype IV GBS disease in Minnesota underscores the value of monitoring prevalent serotypes in a community to detect new epidemiologic trends. In an era when antimicrobial drug prophylaxis is used during childbirth, these findings also highlight the need to be aware of antimicrobial drug–resistant isolates of all serotypes (*26*,*35*). Although maternal immunization with conjugate vaccines incorporating the most common GBS serotypes remains one of the most promising strategies for disease prevention (*27*,*40*), continued assessment of this and other approaches is essential (*8*,*13*,*30*,*37*).

## References

[1] Edwards MS, Baker CJ. Group B streptococcal infections. In: Remington JS, Klein JO, editors. Infectious diseases of the fetus and newborn infant. 5th ed. Philadelphia: Saunders; 2001. p. 1091–156.

[2] Ferrieri P, Wallen L. Neonatal bacterial sepsis. In: Gleason CA, Devaskar S, editors. Avery’s diseases of the newborn. 9th ed. Philadelphia: Elsevier/Saunders; 2012. p. 538–50.

[3] Guilbert J, Levy C, Cohen R; Bacterial Meningitis Group. Delacourt C, Renolleau S, et al. Late and ultra late onset *Streptococcus* B meningitis: clinical and bacteriological data over 6 years in France. Acta Paediatr. 2010;99:47–51. 10.1111/j.1651-2227.2009.01510.x20002014

[4] Imperi M, Gherardi G, Berardi A, Baldassarri L, Pataracchia M, Dicuonzo G, Invasive neonatal GBS infections from an area-based surveillance study in Italy. Clin Microbiol Infect. 2011;17:1834–9 and .10.1111/j.1469-0691.2011.03479.x21722258

[5] Manning SD, Springman AC, Lehotzky E, Lewis MA, Whittam TS, Davies HD. Multilocus sequence types associated with neonatal group B streptococcal sepsis and meningitis in Canada. J Clin Microbiol. 2009;47:1143–8 and .10.1128/JCM.01424-0819158264PMC2668308

[6] Martins ER, Pessanha MA, Ramirez M, Melo-Cristino J; The Portuguese Group for the Study of Streptococcal Infections. Analysis of group B streptococcal isolates from infants and pregnant women in Portugal revealing two lineages with enhanced invasiveness. J Clin Microbiol. 2007;45:3224–9 and .10.1128/JCM.01182-0717699641PMC2045366

[7] Schrag S, Gorwitz R, Fultz-Butts K, Schuchat A. Prevention of perinatal group B streptococcal disease. Revised guidelines from CDC. MMWR Recomm Rep. 2002;51(RR-11):1–22 .12211284

[8] Jordan HT, Farley MM, Craig A, Mohle-Boetani J, Harrison LH, Petit S, Revisiting the need for vaccine prevention of late-onset neonatal group B streptococcal disease: a multistate, population-based analysis. Pediatr Infect Dis J. 2008;27:1057–64 and .10.1097/INF.0b013e318180b3b918989238

[9] Zaleznik DF, Rench MA, Hillier S, Krohn MA, Platt R, Lee M-LT, Invasive disease due to group B streptococcus in pregnant women and neonates from diverse population groups. Clin Infect Dis. 2000;30:276–81 and .10.1086/31366510671328

[10] Harrison LH, Elliott JA, Dwyer DM, Libonati JP, Ferrieri P, Billmann L, Serotype distribution of invasive group B streptococcal isolates in Maryland: implications for vaccine formulation. J Infect Dis. 1998;177:998–1002 and .10.1086/5152609534974

[11] Centers for Disease Control and Prevention. Prevention of perinatal group B streptococcal disease: a public health perspective. MMWR Recomm Rep. 1996;45(RR-7):1–24 .8637497

[12] Phares CR, Lynfield R, Farley MM, Mohle-Boetani J, Harrison LH, Petit S, Epidemiology of invasive group B streptococcal disease in the United States, 1999–2005. JAMA. 2008;299:2056–65 and .10.1001/jama.299.17.205618460666

[13] Verani JR, McGee L, Schrag SJ; Division of Bacterial Diseases, National Center for Immunization and Respiratory Diseases, Centers for Disease Control and Prevention. Prevention of perinatal group B streptococcal disease: revised guidelines from CDC, 2010. MMWR Recomm Rep. 2010;59(RR-10):1–36 .21088663

[14] Gherardi G, Imperi M, Baldassarri L, Pataracchia M, Alfarone G, Recchia S, Molecular epidemiology and distribution of serotypes, surface proteins, and antibiotic resistance among group B streptococci in Italy. J Clin Microbiol. 2007;45:2909–16 and .10.1128/JCM.00999-0717634303PMC2045288

[15] Shet A, Ferrieri P. Neonatal & maternal group B streptococcal infections: a comprehensive review. Indian J Med Res. 2004;120:141–50 .15489551

[16] Diedrick MJ, Flores AE, Hillier SL, Creti R, Ferrieri P. Clonal analysis of colonizing group B *Streptococcus*, serotype IV, an emerging pathogen in the United States. J Clin Microbiol. 2010;48:3100–4 and .10.1128/JCM.00277-1020610684PMC2937746

[17] Slotved H-C, Kong F, Lambertsen L, Sauer S, Gilbert GL. Serotype IX, a proposed new *Streptococcus agalactiae* serotype. J Clin Microbiol. 2007;45:2929–36 and .10.1128/JCM.00117-0717634306PMC2045254

[18] Ferrieri P, Baker CJ, Hillier SL, Flores AE. Diversity of surface protein expression in group B streptococcal colonizing & invasive isolates. Indian J Med Res. 2004;119(Suppl):191–6 .15232193

[19] Johnson DR, Ferrieri P. Group B streptococcal Ibc protein antigen: distribution of two determinants in wild-type strains of common serotypes. J Clin Microbiol. 1984;19:506–10 .620150610.1128/jcm.19.4.506-510.1984PMC271105

[20] Erdogan S, Fagan PK, Talay SR, Rohde M, Ferrieri P, Flores AE, Molecular analysis of group B protective surface protein, a new cell surface protective antigen of group B streptococci. Infect Immun. 2002;70:803–11 and .10.1128/IAI.70.2.803-811.200211796614PMC127721

[21] Ramaswamy SV, Ferrieri P, Madoff LC, Flores AE, Kumar N, Tettelin H, Identification of novel *cps* locus polymorphisms in nontypable group B *Streptococcus.* J Med Microbiol. 2006;55:775–83 and .10.1099/jmm.0.46253-016687599

[22] Amundson NR, Flores AE, Hillier SL, Baker CJ, Ferrieri P. DNA macrorestriction analysis of nontypeable group B streptococcal isolates: clonal evolution of nontypeable and type V isolates. J Clin Microbiol. 2005;43:572–6 and .10.1128/JCM.43.2.572-576.200515695647PMC548113

[23] Benson JA, Ferrieri P. Rapid pulsed-field gel electrophoresis method for group B streptococcus isolates. J Clin Microbiol. 2001;39:3006–8 and .10.1128/JCM.39.8.3006-3008.200111474035PMC88282

[24] Jones N, Bohnsack JF, Takahashi S, Oliver KA, Chan M-S, Kunst F, Multilocus sequence typing system for group B streptococcus. J Clin Microbiol. 2003;41:2530–6 and .10.1128/JCM.41.6.2530-2536.200312791877PMC156480

[25] Feil EJ, Li B, Aanensen DM, Hanage WP, Spratt BG. eBURST: inferring patterns of evolutionary descent among clusters of related bacterial genotypes from multilocus sequence typing data. J Bacteriol. 2004;186:1518–30 and .10.1128/JB.186.5.1518-1530.200414973027PMC344416

[26] Castor ML, Whitney CG, Como-Sabetti K, Facklam RR, Ferrieri P, Bartkus JM, Antibiotic resistance patterns in invasive group B streptococcal isolates. Infect Dis Obstet Gynecol. 2008;2008:727505. 10.1155/2008/727505PMC263736819223967

[27] Edmond KM, Kortsalioudaki C, Scott S, Schrag SJ, Zaidi AKM, Cousens S, Group B streptococcal disease in infants aged younger than 3 months: systemic review and meta-analysis. Lancet. 2012;379:547–56 and .10.1016/S0140-6736(11)61651-622226047

[28] Lin F-YC, Weisman LE, Troendle J, Adams K. Prematurity is the major risk factor for late-onset group B streptococcus disease. J Infect Dis. 2003;188:267–71 and .10.1086/37645712854082

[29] Hamada S, Vearncombe M, McGeer A, Shah PS. Neonatal group B streptococcal disease: incidence, presentation, and mortality. J Matern Fetal Neonatal Med. 2008;21:53–7 and .10.1080/1476705070178747418175244

[30] Van Dyke MK, Phares CR, Lynfield R, Thomas AR, Arnold KE, Craig AS, Evaluation of universal antenatal screening for group B streptococcus. N Engl J Med. 2009;360:2626–36 and .10.1056/NEJMoa080682019535801

[31] Thigpen MC, Whitney CG, Messonnier NE, Zell ER, Lynfield R, Hadler JL, Bacterial meningitis in the United States, 1998–2007. N Engl J Med. 2011;364:2016–25 and .10.1056/NEJMoa100538421612470

[32] Puopolo KM, Madoff LC. Type IV neonatal early-onset group B streptococcal disease in a United States hospital. J Clin Microbiol. 2007;45:1360–2 and .10.1128/JCM.02487-0617267636PMC1865845

[33] Blumberg HM, Stephens DS, Modansky M, Erwin M, Elliot J, Facklam RR, Invasive group B streptococcal disease: the emergence of serotype V. J Infect Dis. 1996;173:365–73 and .10.1093/infdis/173.2.3658568297

[34] Elliott JA, Farmer KD, Facklam RR. Sudden increase in isolation of group B streptococci, serotype V, is not due to emergence of a new pulsed-field gel electrophoresis type. J Clin Microbiol. 1998;36:2115–6 .965097810.1128/jcm.36.7.2115-2116.1998PMC104994

[35] Manning SD, Foxman B, Pierson CL, Tallman P, Baker CJ, Pearlman MD. Correlates of antibiotic-resistant group B streptococcus isolated from pregnant women. Obstet Gynecol. 2003;101:74–9 and .10.1016/S0029-7844(02)02452-312517649

[36] Kothari NJ, Morin CA, Glennen A, Jackson D, Harper J, Schrag SJ, Invasive group B streptococcal disease in the elderly, Minnesota, USA, 2003–2007. Emerg Infect Dis. 2009;15:1279–81 and .10.3201/eid1508.08138119751591PMC2815956

[37] Koenig JM, Keenan WJ. Group B streptococcus and early-onset sepsis in the era of maternal prophylaxis. Pediatr Clin North Am. 2009;56:689–708 and .10.1016/j.pcl.2009.04.00319501699PMC2702484

[38] Luan S-L, Granlund M, Sellin M, Lagergård T, Spratt BG, Norgren M. Multilocus sequence typing of Swedish invasive group B streptococcus isolates indicates a neonatally associated genetic lineage and capsule switching. J Clin Microbiol. 2005;43:3727–33 and .10.1128/JCM.43.8.3727-3733.200516081902PMC1233917

[39] Martins ER, Andreu A, Correia P, Juncosa T, Bosch J, Ramirez M, Group B streptococci causing neonatal infections in Barcelona are a stable clonal population: 18-year surveillance. J Clin Microbiol. 2011;49:2911–8 and .10.1128/JCM.00271-1121697333PMC3147731

[40] Edwards MS, Lane HJ, Hillier SL, Rench MA, Baker CJ. Persistence of functional antibodies to group B streptococcal capsular polysaccharides following immunization with glycoconjugate vaccines. Vaccine. 2012;30:4123–6 and .10.1016/j.vaccine.2012.04.04822537994PMC3392193

# Research article: Serotype IV and Invasive Group B *Streptococcus* Disease in Neonates, Minnesota, USA, 2000–2010^1^

**Authors:** Patricia Ferrieri, Ruth Lynfield, Roberta Creti, Aurea E. Flores

**Affiliations:** University of Minnesota Medical School, Minneapolis, Minnesota, USA (P. Ferrieri, A.E. Flores);; Minnesota Department of Health, St. Paul, Minnesota, USA (R. Lynfield);; Istituto Superiore di Sanità, Rome, Italy (R. Creti)

## Medscape CME Quiz

## Earning CME Credit

To obtain credit, you should first read the journal article. After reading the article, you should be able to answer the following, related, multiple-choice questions. To complete the questions (with a minimum 70% passing score) and earn continuing medical education (CME) credit, please go to www.medscape.org/journal/eid. Credit cannot be obtained for tests completed on paper, although you may use the worksheet below to keep a record of your answers. You must be a registered user on Medscape.org. If you are not registered on Medscape.org, please click on the New Users: Free Registration link on the left hand side of the website to register. Only one answer is correct for each question. Once you successfully answer all post-test questions you will be able to view and/or print your certificate. For questions regarding the content of this activity, contact the accredited provider, CME@medscape.net. For technical assistance, contact CME@webmd.net. American Medical Association’s Physician’s Recognition Award (AMA PRA) credits are accepted in the US as evidence of participation in CME activities. For further information on this award, please refer to http://www.ama-assn.org/ama/pub/category/2922.html. The AMA has determined that physicians not licensed in the US who participate in this CME activity are eligible for *AMA PRA Category 1 Credits*™. Through agreements that the AMA has made with agencies in some countries, AMA PRA credit may be acceptable as evidence of participation in CME activities. If you are not licensed in the US, please complete the questions online, print the certificate and present it to your national medical association for review.

## Article Title:  Serotype IV and Invasive Group B *Streptococcus* Disease in Neonates, Minnesota, USA, 2000–2010

## CME Questions

**1. You are seeing a 2-day-old male infant with fever to 38.3°C and some irritability with poor feeding. You suspect that this child might have group B *Streptococcus* (GBS) infection. Which of the following statements regarding GBS infection among infants is most accurate?**

A. Early-onset (EO) GBS infection is defined by infection between birth and day 6 of life

B. Late-onset (LO) GBS infection is defined by infection between 1 to 3 months of life

C. Screening for GBS in the third trimester has had no effect on the prevalence of EO disease among infants

D. Screening for GBS in the third trimester has primarily reduced the prevalence of LO disease among infants

**2. What should you consider regarding the epidemiology of GBS disease in the current study?**

A. There were more LO vs EO cases of GBS disease

B. Early-onset GBS was almost always associated with cerebrospinal fluid infection

C. Most infants with EO disease were full-term

D. There were no cases of GBS disease after infants were at least 3 months old

**3. What were the most common serotypes of GBS among infected children in the current study?**

A. Ib, II

B. III, Ia

C. IV, Ib

**D. IV, V**

**4. What should you consider regarding infection with serotype IV GBS among children in the current study?**

A. Type IV was associated with higher rates of LO vs EO disease

B. The mortality rate associated with type IV infection was 50%

C. Type IV disease produced higher degrees of fever compared with other serotypes

D. Type IV disease was associated with higher rates of antimicrobial resistance in 2010 compared with other serotypes

## Activity Evaluation

**Table:** 

**1. The activity supported the learning objectives.**				
Strongly Disagree				Strongly Agree
1	2	3	4	5
**2. The material was organized clearly for learning to occur.**				
Strongly Disagree				Strongly Agree
1	2	3	4	5
**3. The content learned from this activity will impact my practice.**				
Strongly Disagree				Strongly Agree
1	2	3	4	5
**4. The activity was presented objectively and free of commercial bias.**				
Strongly Disagree				Strongly Agree
1	2	3	4	5
